# Editorial: Culture and emotion in educational dynamics, volume IV

**DOI:** 10.3389/fpsyg.2026.1837400

**Published:** 2026-04-13

**Authors:** Darío Páez, Silvia da Costa Dutra, Enrique H. Riquelme

**Affiliations:** 1 Universidad Andres Bello, Santiago, Chile; 2 Centro de Investigación, Innovación y Creación, Universidad Católica de Temuco, Temuco, Chile; 3 Departamento de Psicología y Sociología, Universidad de Zaragoza, Zaragoza, Spain; 4 Departamento de Diversidad y Educación Intercultural, Universidad Católica de Temuco, Temuco, Chile

**Keywords:** culture, dynamics, education, emotion, regulation

This new volume of *Culture and emotion in educational dynamics* continues the interdisciplinary dialogue established in previous editions by examining how emotional processes interact with cultural, institutional, and technological dimensions of education. While earlier volumes emphasized the conceptual foundations of this field, the contributions gathered in Volume IV expand the scope of inquiry toward new methodological approaches, emerging learning environments, and the growing attention to wellbeing and emotional development across educational systems.

The studies compiled in this volume reflect the maturation of research on emotions in education. The contributions range from the development of new psychometric tools and computational models for studying emotions to empirical analyses of emotional processes in students, teachers, and educational communities. Moreover, several articles address contemporary transformations affecting education—including digital environments, artificial intelligence, and global communication—while others emphasize the cultural and social contexts through which emotions are experienced and interpreted. The research presented in this volume illustrates the complexity of educational emotions as both individual experiences and socially situated phenomena. The volume is organized around three major thematic blocks that capture the main directions of current research:

(1) Methodological and conceptual advances in the study of emotions in education;
(2) Emotional processes shaping student wellbeing, motivation, and learning; and
(3) Emotional dynamics in educational communities, including teachers, families, and broader cultural contexts.

## Block 1: Advancing the measurement and conceptualization of emotions in education

The first group of studies addresses the need for more refined tools and methodological frameworks to investigate emotional processes in educational settings. As the field expands, developing reliable instruments and innovative analytical approaches becomes essential for capturing the complexity of emotional experiences in learning contexts.

Several contributions focus on the development and validation of measurement instruments. Peng introduces the EpiCT-CI scale, designed to assess epistemic emotions associated with critical thinking and cultural identity construction. The scale integrates emotional dimensions such as curiosity, boredom, and distress in intercultural reflection, offering a novel approach to understanding how emotions influence reasoning processes in culturally diverse contexts. Similarly, Fernández-Berrocal et al. examine the psychometric properties of the Trait Meta-Mood Scale (TMMS-24) in Peruvian university students, confirming its reliability and construct validity as a measure of perceived emotional intelligence. Their findings reinforce the importance of emotional awareness, clarity, and regulation as protective factors in higher education.

Complementing these developments, Wang et al. propose the Adolescent Stressful Academic Events Checklist (ASAEC), a new instrument designed to capture academic stress from an ecological perspective. By combining qualitative interviews with psychometric validation, the study identifies a wide range of stressors emerging from contemporary educational environments, including interactions mediated through digital networks and extended social circles. The scale provides a valuable tool for assessing how academic stress is embedded in increasingly complex social contexts.

Other contributions introduce innovative analytical frameworks for studying emotions in education. Ding et al. employ large-scale social media data to develop the Expectancy–Regulation–Amplification (ERA) model, which conceptualizes academic stress as a dynamic process emerging from the interaction between societal expectations, individual self-regulation capacities, and the amplification effects of digital environments. Their multilayer topic modeling approach illustrates how algorithmic environments can shape the semantic expression of stress and vulnerability among students. Methodological innovation is also evident in Zhang, Cai, et al.'s work on the Dynamic Microexpression Training Tool (DMETT). Moving beyond traditional static recognition methods, the authors design a training system that allows participants to identify subtle emotional expressions in dynamic contexts. Their findings demonstrate that targeted training significantly improves individuals' ability to recognize microexpressions, providing new possibilities for studying emotional perception and communication.

These studies demonstrate how the field is increasingly combining psychometric rigor, computational methods, and experimental approaches to deepen the understanding of emotions in educational contexts. By refining measurement and modeling techniques, they provide essential methodological foundations for the research presented in the following sections of the volume.

## Block 2: Student emotional processes, wellbeing, and learning

The second block of contributions examines the emotional mechanisms that shape students' engagement, wellbeing, and academic performance. Across diverse educational contexts, these studies highlight the ways in which emotions interact with motivation, resilience, and social relationships to influence learning trajectories. A number of studies explore the role of emotional resources in academic adaptation and success. Zhou et al. investigate the relationship between fear of academic failure and creative problem-solving among university students, demonstrating that curiosity and grit mediate this association. Their findings suggest that while fear may initially hinder performance, the presence of motivational resources such as perseverance can transform emotional tension into productive engagement with complex tasks.

In a similar vein, Shaoshuai et al. examine how psychosocial competencies—including emotional quotient, adversity quotient, and psychological resilience—contribute to academic engagement among students in Traditional Chinese Medicine programs. The results show that emotional competencies not only support academic persistence but also interact with family relationships to stabilize students' responses to academic challenges. The importance of emotional processes is also emphasized in research on academic stress and mental health. Huang et al. analyze the effects of mindfulness-based stress reduction programs using a dual-factor model of mental health, showing that mindfulness interventions can simultaneously enhance positive wellbeing and reduce psychological distress among university students. Complementing this perspective, Qi et al. conduct a meta-analysis examining the impact of achievement emotions in online learning environments. Their findings indicate that positive emotions are moderately associated with better academic performance, while negative emotions tend to hinder learning outcomes.

Other studies investigate the broader relationship between education and wellbeing. Dong et al.'s meta-analysis of 59 empirical studies demonstrates that educational attainment is positively related to subjective wellbeing, although the strength of this relationship varies across rural and urban contexts. These findings underscore the social dimension of emotional wellbeing and its connection to educational opportunities.

A distinctive feature of this volume is the attention given to emerging learning environments and technological transformations. Pan and Li explore learner wellbeing in AI-integrated language learning environments, demonstrating that perceived teacher support and growth language mindsets positively influence student wellbeing through attitudes toward generative artificial intelligence. Zhao et al. further examine how academic buoyancy supports engagement in English speaking learning, showing that enjoyment mediates the relationship between resilience and participation while anxiety moderates these effects.

Other contributions analyze emotional experiences associated with language learning in contemporary social contexts. Tian et al. investigate how social appearance anxiety and learning autonomy influence collaborative dynamics in second language acquisition, highlighting the importance of supportive learning environments for reducing psychological barriers. Similarly, Zhang, Wu, et al. examine the use of English on social media and its influence on interpersonal communication intentions among university students. Their results suggest that language confidence and cultural identity play important mediating roles in shaping communication behaviors. Finally, Zhan and Cheng analyze the impact of multisensory language learning models, demonstrating that integrated visual, auditory, and tactile stimuli can simultaneously improve language skills and reduce learning anxiety. Together, the studies in this block illustrate how emotional experiences shape learning processes across both traditional and technologically mediated educational settings.

## Block 3: Emotional dynamics in educational communities and cultural contexts

The final group of articles expands the perspective from individual learners to the broader social environments in which education takes place. These studies explore how emotions circulate within classrooms, institutions, families, and cultural communities.

Several contributions focus on the emotional dimensions of teaching and classroom interaction. Bi and Rauduvaite analyze how self-efficacy develops among prospective music teachers through singing-related coursework, emphasizing the role of supportive pedagogical environments and collaborative learning. Yin and Zhang examine the factors influencing professional leadership among university teachers, demonstrating that personal qualities and institutional culture contribute to leadership development through the mediating role of self-efficacy.

Classroom climate and emotional regulation also emerge as key themes. Muñoz-Troncoso and Riquelme explore how physical classroom conditions affect teachers' perceptions of classroom climate and personal wellbeing, revealing that factors such as lighting, space, and temperature influence both teacher–student relationships and the overall emotional environment of the classroom. Complementing this perspective, Alarcón-Espinoza et al. investigate emotional self-regulation in classroom conflicts through systematic observation and sequential analysis. Their results highlight the importance of teachers' role in recognizing and managing emotional expressions during classroom interactions. Liu and Demetriou analyze how play-based learning activities can help young children understand anger within a Chinese kindergarten context. Their findings suggest that culturally adapted pedagogical strategies can promote emotional literacy and social interaction among young learners.

Beyond classroom contexts, several articles emphasize the cultural and social dimensions of emotional experience. Rincón-Unigarro et al. analyze the emotional dynamics of participation in the Colombian Carnaval de Negros y Blancos, demonstrating how collective rituals generate experiences of awe, emotional synchrony, and shared identity. Their results show that collective effervescence strengthens community identification and promotes prosocial behaviors. Cultural values also shape educational motivation. Lin et al. investigate how collectivist cultural orientations and family expectations influence goal orientation and mathematics achievement among vocational students. Their findings reveal the complex interplay between cultural values, motivation, and academic outcomes.

Finally, Uǧurlu et al. explore the experiences of mothers navigating the school entry process for children with hearing loss. Through qualitative interviews, the study highlights the emotional challenges families face when seeking educational support and the importance of accessible institutional resources. These findings underscore the central role of family networks and social support systems in shaping educational experiences.

## Concluding reflections

The studies gathered in Volume IV illustrate the growing diversity and methodological sophistication of research on emotions in education. Across different contexts and disciplines, the contributions highlight how emotional experiences influence learning processes, institutional dynamics, and cultural participation.

Three overarching trends emerge from the volume. First, the field is increasingly investing in innovative measurement tools and analytical frameworks, enabling more precise investigation of emotional processes. Second, there is a growing emphasis on student wellbeing and psychological resources, reflecting heightened awareness of the emotional demands of contemporary education. Finally, the research underscores the importance of social and cultural contexts, demonstrating that emotions in education are not solely individual experiences but are embedded in collective practices, institutional environments, and cultural traditions.

By bringing together these diverse perspectives, this volume contributes to a deeper understanding of how emotions and culture interact within educational dynamics. Ultimately, the research presented here supports the development of more inclusive, responsive, and emotionally aware educational systems capable of addressing the challenges of an increasingly complex world.

